# Early and sensitive diagnosis of celiac autoimmune disease by using carboxylic acid functionalized magnetic nanoparticles-assisted biosensing platform

**DOI:** 10.1007/s00604-025-07129-6

**Published:** 2025-04-08

**Authors:** Elif Burcu Aydın, Muhammet Aydın, Mustafa Kemal Sezgintürk

**Affiliations:** 1 Scientific and Technological Research Center, Tekirdağ NamıK Kemal University, Tekirdağ, Turkey; 2 Bioengineering Department, Faculty of Engineering, Çanakkale Onsekiz Mart University, Çanakkale, Turkey

**Keywords:** Anti-tissue transglutaminase antibody, Magneto biosensor, 3-phosphonopropionic acid, Magnetic beads

## Abstract

A novel impedimetric magneto-immunosensor based on iron oxide (Fe_3_O_4_) nanoparticles coated with 3-phosphonopropionic acid (3-PPA) (functionalized magnetic beads, or FMBs) was created for the highly sensitive and selective detection of anti-tissue transglutaminase antibody (anti-tTG) in human serum. This label-less immunosensor was introduced by magnetically attaching FMBs onto the working electrode surface with a neodium magnet. The FMBs were utilized as a sensing interface and had carboxylic acid groups for tTG molecules, which could selectively link the target anti-tTG antibody. The FMBs modification steps were carried out in microcentrifuge tubes and concentrated with magnetic force before electrochemical analyses. The specific immuno-interactions on the FMBs surface were characterized by using the electrochemical and microscopic techniques, and in the presence of anti-tTG antibodies, they were captured by tTG-immobilized magnetic beads, and significant increases were observed in impedimetric response. The magneto biosensor response was linearly related to the anti-tTG antibody level in a broad linear range of 0.125–15.62 U/mL and a low detection limit (LOD) of 0.04 U/mL. Additionally, this magneto sensor was stable, repeatable, reproducible, selective, and sensitive for determination of the anti-tTG. The commercial enzyme-linked immunosorbent assay (ELISA) method was employed to compare the responses of the suggested immunosensor in actual samples. The magneto biosensor results were in good agreement with the ELISA reference technique results. Consequently, the biosensor performance in the analysis of serum samples was acceptable.

**Graphical Abstract:**

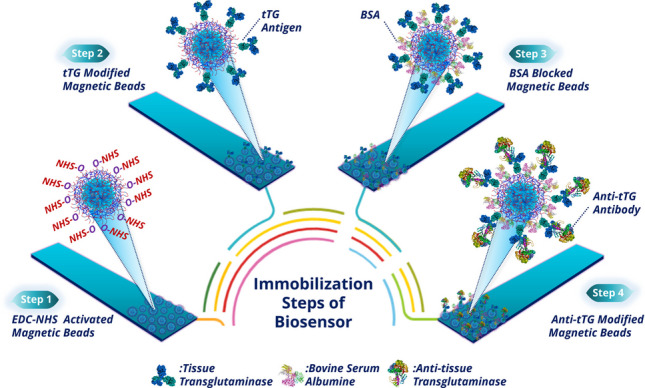

**Supplementary Information:**

The online version contains supplementary material available at 10.1007/s00604-025-07129-6.

## Introduction

Celiac disease (CsD) is an immune-mediated condition with a prevalence of 1% in the general population, 1.5% in children, and 2.7% in the elderly. Some CsD patients may not exhibit any symptoms for years, and the disease’s clinical manifestations range from mild to severe [1, 2]. This disease is caused by a reaction to gluten, a protein composed of gliadins and glutenins that is present in wheat, barley, and rye. When a person with celiac disease eats gluten, their immune system targets the lining of their small intestine, resulting in a variety of symptoms [3, 4]. Since CsD primarily affects and destroys the mucosa of the upper small intestine, a conclusive diagnosis of CsD requires recurrent intestinal biopsy (usually three to five times) and tissue histopathologic evaluation. However, because a biopsy is invasive, it cannot be used frequently or routinely [5]. Anti-gliadin, anti-deamidated gliadin, anti-endomysial, and anti-tTG are CsD-specific antibodies that are generated in the intestinal mucosa upon gluten exposure and manifest in the diseased intestinal mucosa, saliva, and blood [6]. Although a small intestinal biopsy is still the gold standard for diagnosing CsD, serological screening tests that identify these autoantibodies have grown more sensitive and specific, and it is anticipated that the official criteria for celiac disease will soon be modified to include both genetic and serological testing [7]. In addition, the degree of villous atrophy is correlated with the high amounts of anti-tTG antibodies, which are highly specific and sensitive for CsD [8]. Moreover, since anti-gliadin antibodies have lower sensitivity and specificity, the anti-tTG test is now the serological test routinely employed [9]. If the amount of anti-tTG in human serum is < 12 U/mL, 12–18 U/mL, and > 18 U/mL, it is negative, borderline positive, and positive, respectively [10]. Kamilova et al. (2022) investigated the anti-tTG level in children’s serum samples, and the level of anti-tTG antibodies in the CsD and healthy persons was 171.7 ± 20.03 U/mL (*n* = 76) and 5.2 ± 2.6 U/mL (*n* = 32), respectively [11]. Ajdani et al. (2022) investigated the salivary anti-tTG amount in saliva samples, and the anti-tTG concentration in the CsD and healthy control groups was 1822.37 ± 2232.81 U/mL (*n* = 39) and 95.04 ± 183.20 U/mL (*n* = 39), respectively [12].

Serological analysis methods based on the ELISA are frequently used in regular laboratory testing for CsD screening [7, 13]. These techniques are also time-consuming, costly, and complex. To address these issues, electrochemical immunosensors—a straightforward, extremely sensitive, non-invasive, quick-reaction, and inexpensive method—are a good choice [14–16]. Because of its great analytical performance, ease of use, portability, and affordability, electrochemical detection is one of the most widely used biosensing modalities [17–19]. A number of quick, easy, and affordable tests have been created as a first step in expediting the diagnosis of CsD in the doctor’s office due to the high frequency of the disease and the negative effects of delayed detection [17, 20].

Electrochemical impedance spectroscopy (EIS) is an effective and instructive method for assessing the interfacial characteristics of biochemical and biophysical processes [21, 22]. Additionally, EIS has become more and more successful in designing and developing sensor systems. Furthermore, because of its adaptability, EIS may be used to monitor and regulate the various steps required for the sensor’s construction and final characterization. The analyte concentration can be determined using this technique, which has also been used to monitor changes in the electrical properties of the sensor system when the concentration of the analyte of interest increases [23]. The fact that the stimulus sinusoidal voltage is low and does not harm or disrupt the majority of biorecognition layers is an important benefit of EIS biosensors [24].

Magnetic Fe_3_O_4_ nanoparticles are appealing nanomaterials in the field of material science, chemistry, and physics owing to their advantageous characteristics, such as superparamagnetic feature, good biocompatibility, low toxicity, and easy-to-prepare and use functions [25, 26]. These nanoparticles can interact with cells, viruses, proteins, nucleic acids, and other substances because of their controlled size. Proper surface modification of these nanoparticles can enable their use in different biological applications like drug delivery, biosensors, and nanoelectronics [27]. The ability of these particles to improve analytical selectivity and sensitivity by facilitating electron transport and enhancing electrode conductivity has led to their increasing use in numerous electrochemical applications. The capacity to create a proper microenvironment where biomolecules can directly exchange electrons with an electrode is another essential characteristic of these nanoparticles [28]. Because magnetic silica nanoparticles are biocompatible, readily renewable, and durable against degradation, they are of tremendous interest in current research as unique immobilizing carriers of biomolecules [29]. In addition to shielding the nanoparticles in solution from oxidation, the silica outer shell and inner iron-oxide core offer locations for surface functionalization with different biological ligands in biomedical applications [30, 31]. In addition, silica has a non-magnetic shell that can reduce the strength of the magnetic bipolar connection between particles and is chemically inert [32]. 3-PPA, an organophosphonate coupling agent, has found numerous uses in surface ornamentation by producing a monolayer carrying carboxyl ends. Since the active carboxyl groups (-COOH) simplify further modification and can form covalent bonds with other active ends, like the amino (-NH_2_), which can easily conjugate with biomolecules, this layer with carboxyl ends offers an active platform for further bio-modification or connecting organic molecules onto the nanoparticles. In addition, the carboxyl-organophosphonate-shelled modified magnetic nanoparticles are injectable, biocompatible, and non-toxic [33]. Therefore, 3-PPA is an attractive candidate for Fe_3_O_4_ nanoparticles modification.

In this study, a highly sensitive strategy based on FMBs was developed for the detection of anti-tTG antibody. Magnetic Fe_3_O_4_ nanoparticles were attached to the sensing surface via magnetic force, and they increased the loading of biomolecules. In addition, 3-PPA-functionalized beads provided a proper microenvironment to retain the bioactivity of the tissue transglutaminase biorecognition molecules and effectively promoted electron transfer. The use of this strategy during the design of the biosensor contributed to the high sensitivity of the suggested immunosensor, in addition to a lower LOD value. These superiorities made FMBs a proper choice for designing ultra-sensitive biosensors. This suggested magneto sensor illustrated a wide linear range and an ultralow LOD for anti-tTG antibody. Moreover, this novel magneto biosensor had outstanding stability and efficacy in the examination of human saliva and serum samples, suggesting that it may find application in clinical diagnosis. The results obtained using this biosensor and those obtained using ELISA were compared, and a good correlation was achieved between the two methods.

## Experimental section

### Materials and apparatus

The reagents and chemicals were analytical-grade and employed without any alterations. Potassium hexacyanoferrate (II) (K_4_[Fe(CN)_6_]), potassium hexacyanoferrate(III) (K_3_[Fe(CN)_6_]), iron (II) chloride tetrahydrate (FeCl_2_.4H_2_O), iron (III) chloride tetrahydrate (FeCl_3_.4H_2_O), 3-PPA, tetraorthosilicate (TEOS), ammonia water (NH_4_OH), 1-Ethyl-3-(3-dimethylaminopropyl) carbodiimide (EDC), N-Hydroxysuccinimide (NHS), potassium chloride (KCl), indium tin oxide (ITO) sheet, bovine serum albumin (BSA), tTG, anti-tTG antibody, interleukin 1α (IL1α), interleukin 1β (IL1β), interleukin 8 (IL8), and high mobility group box 1 (HMGB1) were from Sigma-Aldrich (St. Louis, USA). Ammonia solution (25%) was obtained from Merck (Darmstadt, Germany). Phosphate buffer saline (PBS) with a pH of 7.4 was prepared using potassium phosphate dibasic (K_2_HPO_4_) and potassium phosphate monobasic (KH_2_PO_4_), obtained from Merck (Darmstadt, Germany). 2-(N-Morpholino) ethanesulfonic acid (MES) was obtained from Acros Chemicals (Belgium). The anti-tTG antibody ELISA kit was provided by BT Lab (Birmingham, England).

The EIS/CV and DPV/SWV electrochemical evaluations were performed using a Gamry Reference 1000 (Warminster, USA) and Metrohm Autolab AUT204 potentiostat (Utrecht, The Netherlands) connected to a three-electrode cell, respectively. A classical three-electrode system was adopted, including an ITO sheet as a working electrode, an Ag/AgCl reference electrode (BASi, USA), and a platinum wire auxiliary electrode (BASi, USA). All electrochemical experiments were done by employing a 5 mM [Fe(CN)_6_]^3−/4−^ containing a 0.1 M KCl solution. For the CV measurement, the potential was scanned from − 0.5 to 1 V with a scan rate of 0.1 V/s. EIS measurements were done in the frequency range of 0.5 Hz–50 kHz and a voltage of 5 mV amplitude at the formal potential of 0.276 V. For SWV measurement, the potential was scanned between 0 and 1.0 V. The interval time, modulation amplitude, and scan rate of SWV were 0.04 s, 0.02 V, and 0.125 V/s, respectively. For DPV measurement, the potential was recorded between 0 and 0.8 V. The interval time, modulation amplitude, and step potential of DPV were 0.5 s, 50 mV, and 5 mV, respectively. An Orion Star™ A111 (Thermo Scientific, USA) benchtop pH meter was used for pH regulation. Field emission scanning electron microscope (FE-SEM, Quanta FEG 250, USA), energy dispersive X-ray spectroscopy (EDS, USA), Fourier transform infrared spectroscopy (FTIR, Bruker Optics, Germany), and X-ray diffraction (XRD, PANalytical Empyrean, UK) studies were performed for morphological and structural investigation.

### Procedures

#### Synthesizing of Fe3O4 nanoparticles

Fe_3_O_4_ nanoparticles were synthesized by the earlier protocol reported by the conventional chemical precipitation method. Iron salts of FeCl_3_.6H_2_O and FeCl_2_.4H_2_O in a 2:1 ratio were dissolved in ultrapure water. Then, this mixture was stirred homogeneously, and NH_4_OH (26%, 10 mL) was added rapidly to maintain pH 10. After heating the mixture to 80 °C, the black precipitate was produced. The collected precipitate was thoroughly washed with ultrapure water and dried at 80 °C.

### Preparation of carboxylic acid-functionalized silica-coated FMBs

The magnetic silica nanoparticles were constructed according to Stöber’s functionalization process [34]. First of all, 100 mg of magnetic nanoparticles was weighed and transferred to a round-bottom flask, and 40 mL of ethyl alcohol and 10 mL of water were added. After that, this mixture was sonicated for 30 min. Then, 2.5 mL of TEOS and 1.25 mL of ammonia were added to this mixture to generate hydroxyl ends on the particles and mixed overnight at room temperature. A permanent magnet was then used to separate them from the mixture, and they were then cleaned using ultrapure water and a lot of ethanol. The magnetic silica nanoparticles were sonicated in ultrapure water for 10 min, and then they were stirred in 50 mM 3-PPA aqueous solutions overnight. After that, a persistent magnetic field was used to separate the altered magnetic nanoparticles, and ultrapure water was used to wash them.

### Anti-tTG antibody-specific FMBs fabrication procedure

The anti-tTG antibody-specific FMBs were prepared in three stages. In brief, the FMBs (10 µL) were quickly moved into 0.5 mL centrifuge tubes and activated with NHS/EDC (4 mM:1 mM, 20 µL) by stirring with a vortex mixer at 950 rpm for 30 min. Then, activated magnetic beads were separated and rinsed twice with 50 µL of MES (50 mM, pH 6). Subsequently, 20 µL of transglutaminase (38 ng/mL) solution was added onto the activated FMBs and stirred at 950 rpm for 30 min under room conditions. After stirring, the tTG-immobilized FMBs were separated and washed with MES buffer (50 mM, pH 6) to get rid of any physically bound transglutaminase molecules. Then, the tTG-immobilized FMBs were stirred at 950 rpm for 30 min in BSA (1%) blocking solution followed by washing with MES buffer (50 mM, pH 6). Subsequently, they were ready to detect anti-tTG antibodies, and for this purpose, anti-tTG antibody solution was added to the centrifuge tube containing tTG-immobilized FMBs for the covalent conjugation of tTG and anti-tTG antibody molecules, and this centrifuge tube was stirred at room temperature for 30 min. After each modification step, the constructed magnetic beads were separated with magnetic force, and electrochemical measurements were performed by means of a disposable ITO electrode.

### Fabrication of anti-tTG antibody-specific magneto biosensor

The ITO sheet was ultrasonically washed with acetone, soap solution, and ultrapure water for 5 min each, followed by successive treatment in an activation solution of H_2_O_2_:NH_4_OH:H_2_O (1:1:5) for 60 min. To construct the anti-tTG biosensor, FMBs were fixed to the conductive side of the ITO sheet via the magnetic force of magnetic beads and neodium magnet, and for this purpose, a magnet was placed below the ITO sheet. The magnet under the electrode prevented the magnetic nanoparticles from flowing from the electrode surface into the electrolyte solution. In this work, the FMBs were selected as an immune sensing platform for immobilizing tTG proteins, which were specific for anti-tTG antibodies, and before electrochemical analyses, the washed nanoparticles were concentrated using a magnet. The electrochemical behavior of the modified magneto biosensor was further studied by EIS, CV, DPV, and SWV techniques, and the analysis results were recorded.

### Detection of anti-tTG antibody

The anti-tTG antibody-specific FMBs were transferred to the centrifuge tubes and stirred in 20 µL of a different concentration of standard anti-tTG antibody solution for 30 min at 25 °C. After stirring, the anti-tTG-immobilized FMBs were separated and washed with MES buffer (50 mM, pH 6) to get rid of any physically bound anti-tTG molecules. Prior to and following the specific binding between the antigen and antibody, the EIS signals were evaluated in ferri-ferro redox solution. The biorecognition event was monitored by drawing a calibration curve between the immunosensor signal and the anti-tTG antibody concentration.

### Biological sample analysis

To assess the recommended magneto biosensor’s suitability, serum samples were purchased from Sigma-Aldrich (St. Louis, USA) and these samples were utilized after fivefold dilution of samples with PBS buffer. These biological samples were spiked with different anti-tTG antibody concentrations (1.25 and 12.5 U/mL), diluted (1:5), and analyzed.

## Results and discussion

**Scheme 1:**
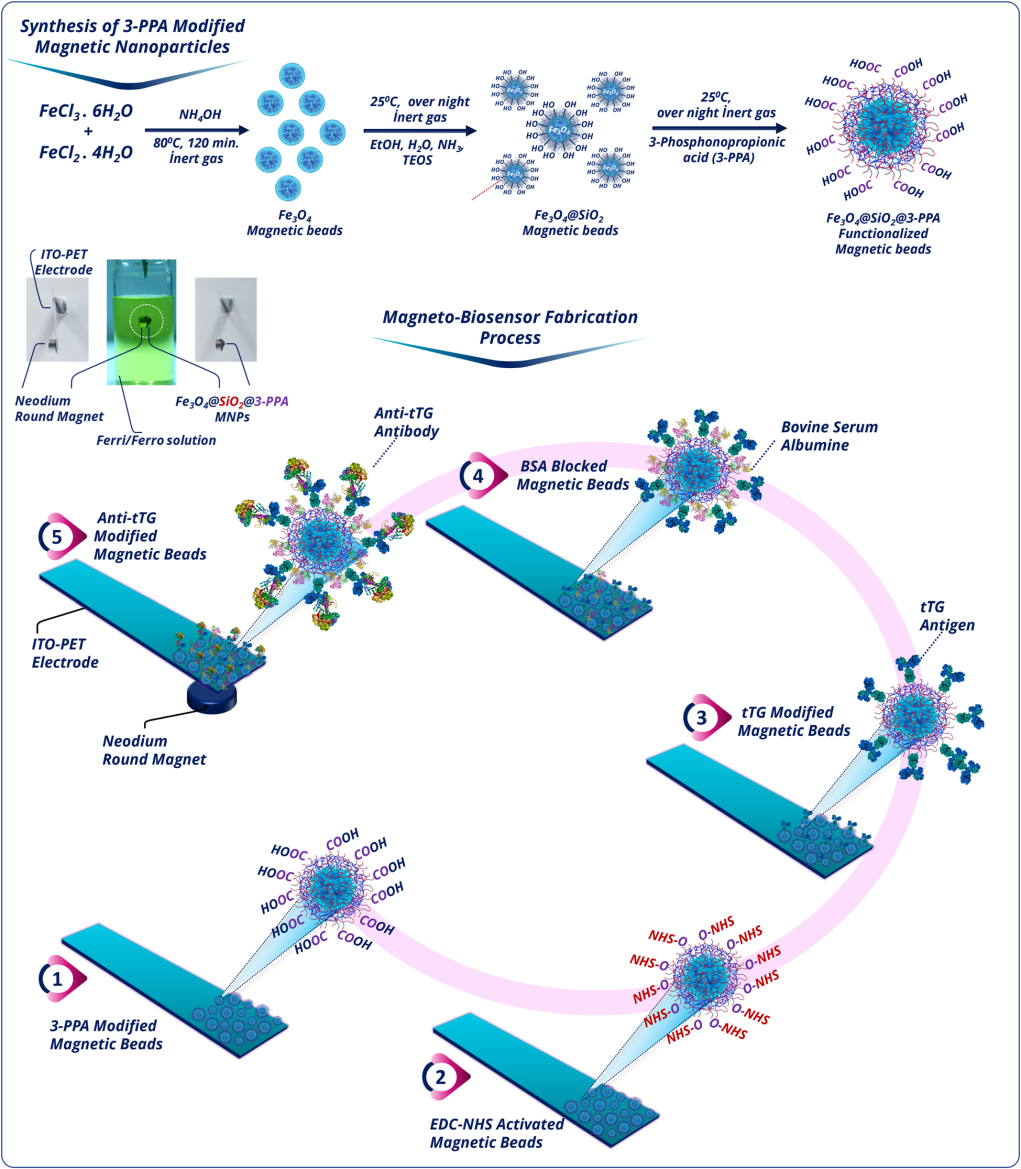
Preparation procedures of FMBs and schematic illustration of the construction process of the label-free anti-tTG magneto biosensor

Here, novel FMBs were successfully produced and used to construct an anti-tTG antibody immunosensor as illustrated in Scheme 1.

The FMBs were employed as the base owing to their good conductivity and biocompatibility. First, the pure Fe_3_O_4_ nanoparticles and magnetic silica nanoparticles were synthesized by well-known co-precipitation and Stöber’s methods, respectively. Then, 3-PPA-terminated with carboxylic acid group reagent was directly applied to the surface of Fe_3_O_4_@SiO_2_ by phosphonate bond formation between phosphonic acid end groups of 3-PPA and hydroxyl groups of Fe_3_O_4_@SiO_2_ nanoparticles. 3-PPA as an immobilization matrix for biomolecules had an important ability to produce self-assembled monolayers on the metal oxide surfaces, and it had a good stability in aqueous solutions. Thus, 3-PPA formed a dense monolayer to immobilize tTG antigens and supported specific biorecognition of target analytes. Before the biomolecules were immobilized on the FMBs, they were reacted with EDC/NHS to activate the carboxylic acid ends of 3-PPA for the formation of a more effective covalent peptide bond between the primary amino group of tTG and the carboxylic acid groups of 3-PPA. Subsequently, the tTG-immobilized FMBs were treated with BSA to block the free active carboxylic acid ends present on the FMBs and avoid non-specific adsorption. Finally, the prepared magneto biosensor was utilized for the attachment of capturing anti-tTG antibody.

### Characterization of the different nanoparticles

The morphology, size, and chemical structure of Fe_3_O_4_, Fe_3_O_4_@SiO_2_, and Fe_3_O_4_@SiO_2_@3-PPA were successfully characterized by FTIR (Fig. SI−1), Raman (Fig. SI−2), XRD (Fig. SI−3), SEM (Fig. SI−4), and EDS (Fig. SI−5) one by one.

**Fig. 1:**
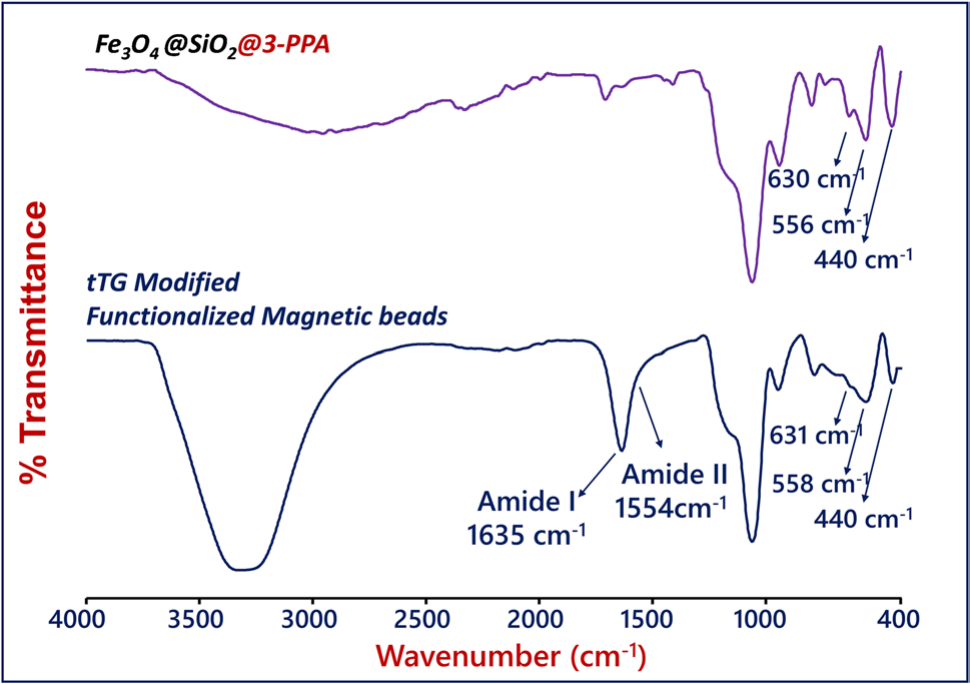
FTIR spectra of FMBs and tTG-attached FMBs

The successful coating of 3-PPA on the Fe_3_O_4_@SiO_2_ particles was also followed by performing FTIR measurements. Figure 1 represents the FTIR spectra of carboxylic acid-functionalized and tTG-attached nanoparticles. The FTIR spectrum of FMBs displayed a strong absorption band corresponding to carbonyl vibrations that appeared at about 1700 cm^−1^. The FTIR spectrum of tTG-attached FMBs exhibited characteristic bands at 1635 cm^−1^ (amide I) and 1554 cm^−1^ (amide II), which were owing to protein molecules on the surface of nanoparticles. The changes in FTIR spectra demonstrated the successful coating of tTG on the FMBs and anti-tTG-specific surface fabrication [35].

### Electrochemical behavior of magneto anti-tTG immunosensor

**Fig. 2:**
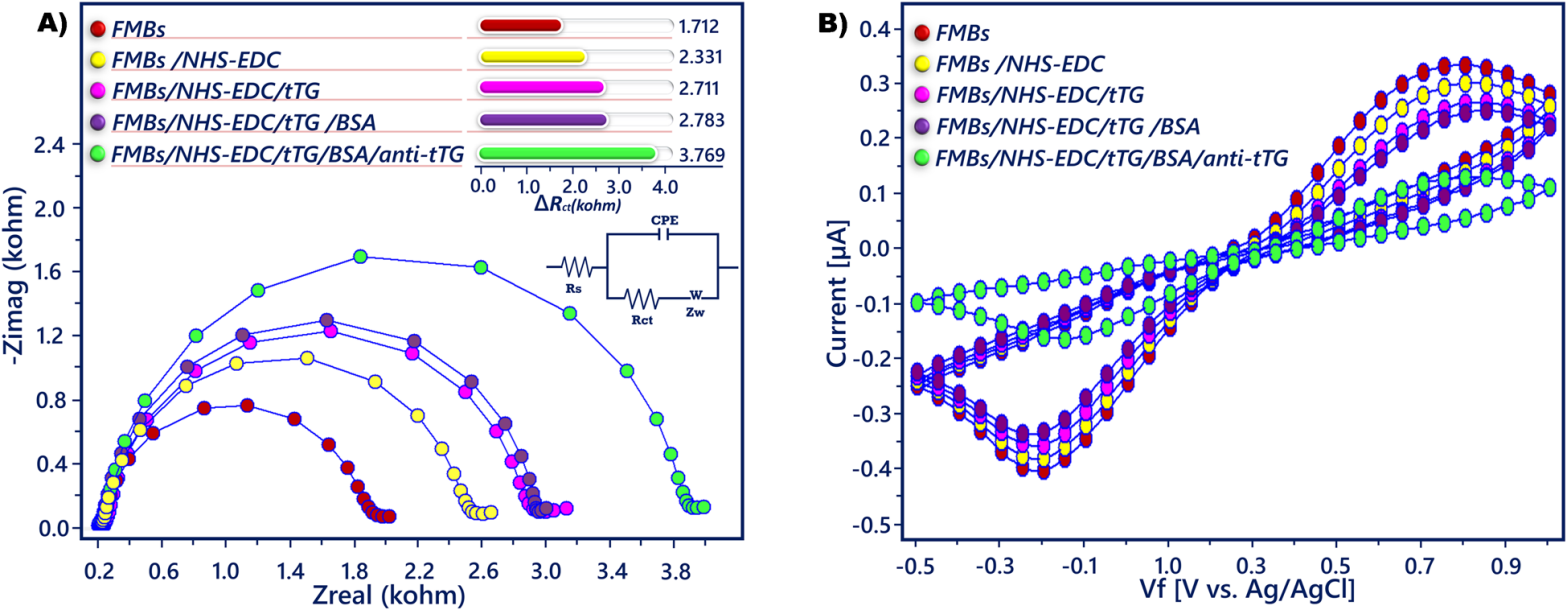
**A** EIS and **B** CV responses recorded in redox solution (5.0 mM K_3_Fe(CN)_6_/K_4_Fe(CN)_6_) during biosensor fabrication. Randless equivalent circuit (**A**, inset)

**Fig. 3:**
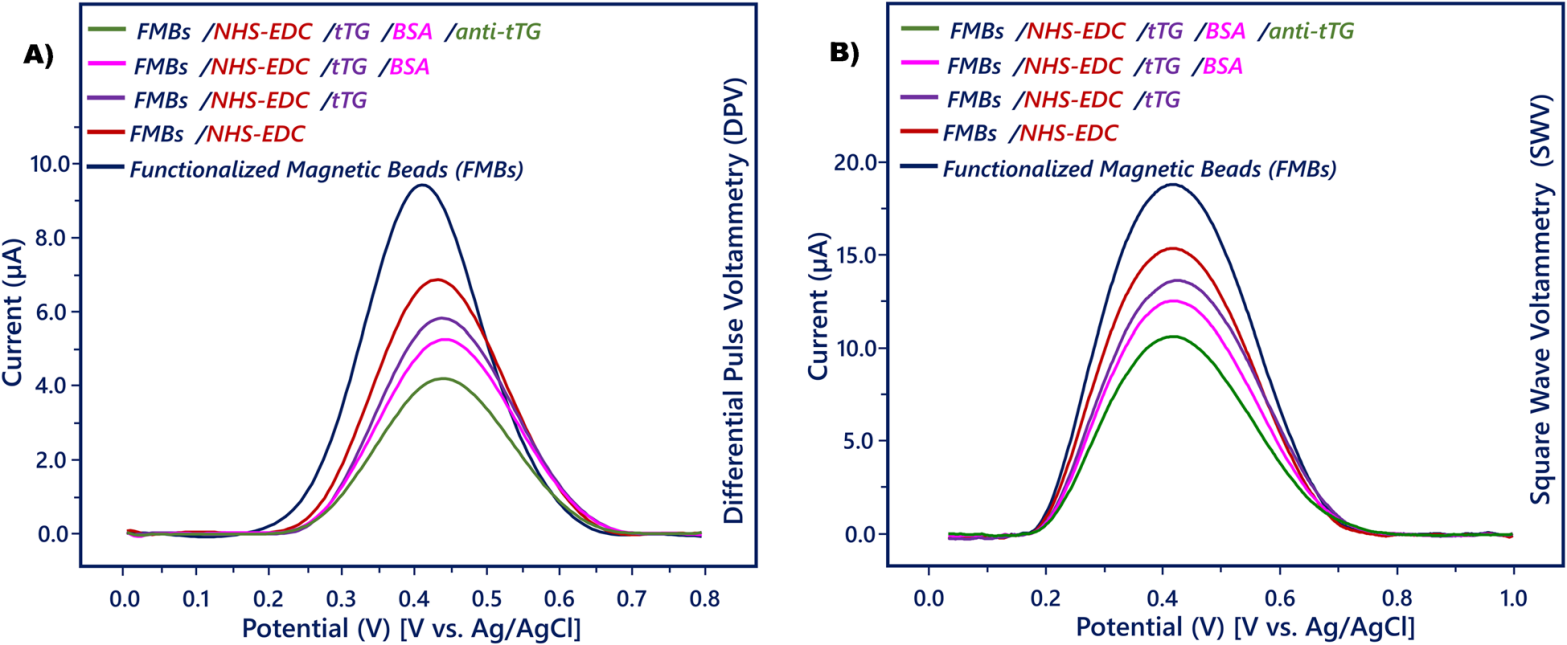
**A** DPV and **B** SWV responses recorded in redox solution (5.0 mM K_3_Fe(CN)_6_/K_4_Fe(CN)_6_) during biosensor fabrication

EIS is a useful and simple technique for describing the immunosensors’ sequential manufacturing process [36]. The electrochemical properties of the anti-tTG magneto biosensor were examined by EIS, CV, DPV, and SWV measurements (Figs. 2, 3, and SI−6). In order to perform electrochemical measurements, prepared magnetic beads were easily attached to the ITO sensing surface via magnetic force, and a neodium magnet was positioned on the back of the ITO sheet. The analysis conditions of the magneto biosensor prepared for anti-tTG measurement were described previously.

The interface characteristics of functionalized sensing surfaces have also been described using EIS. In order to interface modeling Randles equivalent circuit, which includes the solution resistance (*R*_s_), Warburg impedance (*Z*_w_), constant phase element (CPE), and the charge transfer resistance (*R*_ct_), is usually utilized. It is commonly recognized that the impedance plot’s high-frequency region displays a semicircle associated with the ferri-ferro redox couple, while the low-frequency region displays a Warburg line that represents the process’s diffusion step. The *R*_ct_ of different functionalized electrodes can be used to calculate the semicircle diameter [37, 38].

The Nyquist diagrams in Fig. 2A illustrate the EIS spectra of different FMBs. These recorded EIS spectra were fitted in a Randles circuit to extract the value of *R*_ct_. The FMBs-attached electrode surface had a small Nyquist diagram and also a low *R*_ct_ (1712 Ω) because of the good conductivity of FMBs that could facilitate electron transfer between the FMBs surface and the electroactive species in the redox probe. After the carboxylic groups’ activation procedure, an increase was observed in *R*_ct_ (2331 Ω) due to the repulsion effect between the negative groups of 3-PPA and the redox couple. Immobilization of tTG on FMBs increased the EIS signal (2711 Ω), because the biomolecule acted as an insulating layer and prevented the diffusion of the redox probe toward the sensing surface. Similarly, by blocking the free carboxylic acid ends with BSA, increases were observed in Nyquist plot diameter and *R*_ct_ (2783 Ω). When the BSA-attached FMBs were incubated with anti-tTG antibody, the Nyquist plot diameter and *R*_ct_ (3769 Ω) increased due to specific immunoreaction between tTG and anti-tTG. This reaction caused an immunocomplex hydrophobic protein layer production on the FMBs surface.

The stepwise assembly on the FMBs was also monitored by CV measurement in the presence of redox couple solution (Fig. 2B). The FMBs-attached electrode had a representative CV curve due to the good electrical conductivity of FMBs. There were decreases in the peak currents when the FMBs were activated with NHS/EDC. The low conductivity of the protein, which inhibited electron transfer, caused declines in the CV peak currents following the immobilization of tTG. Then, the CVs signal further decreased when the FMBs were stirred in the BSA solution. Similarly, by capturing the anti-tTG antibodies with tTG antigens, anodic and cathodic peaks were further decreased due to the insulating character of biomolecules. The similar results of EIS and CV confirmed the successful production of the immunosensor.

The manufacturing of anti-tTG-specific FMBs and immunoaffinities between tTG and anti-tTG was also analyzed with DPV and SWV measurements. The DPV (Fig. 3A) and SWV (Fig. 3B) peak currents were high due to the good electron transfer property of FMBs. When activated FMBs were attached to the electrode surface, the current heights decreased. After the activated FMBs were incubated with tTG, the lower current responses were recorded because of the large resistance of biomolecules to electron transfer. When the free carboxylic acid ends were modified with BSA, decreases in the peak currents were observed, which proved the electron-transfer barrier formation. The immunoreaction between the tTG and anti-tTG formed an immunocomplex and hindered electron transfer by the formation of an insulating film of protein molecules.

### SEM analysis

**Fig. 4:**
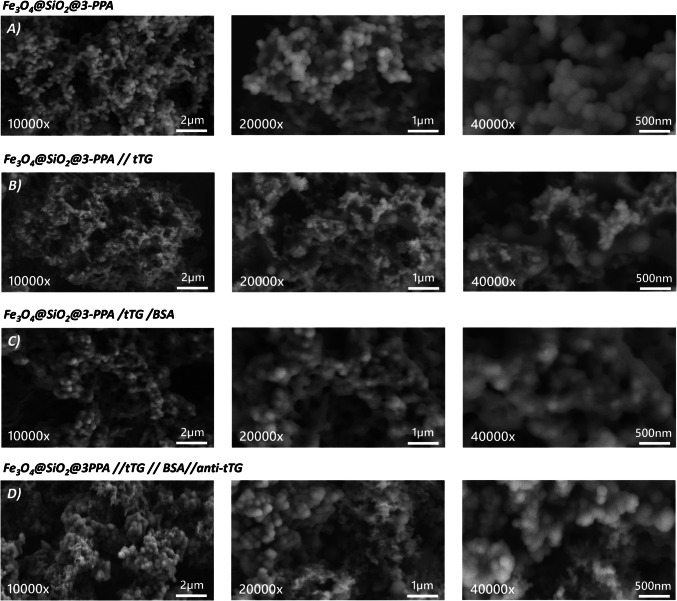
SEM images of FMBs (**A**), tTG (**B**), BSA (**C**), and anti-tTG (**D**) attached-magnetic beads)

SEM analysis was performed on the modified magnetite nanoparticles in order to determine the changes in their size and morphology during the functionalization. As seen in Fig. 4, the size of the magnetic nanoparticles increased due to the attachment of biomolecules on the FMBs surface. Figure 4A shows the SEM images obtained for FMBs, and this figure demonstrates that they were spherical and homogenous in structure and monodispersed. Additionally, the FMBs had a larger surface, which introduced a larger surface area for the attachment of tTG. In the case of the tTG antigen-attached image from Fig. 4B, it was noticed that the immobilization of tTG to the FMBs resulted in protein aggregation due to covalent binding of tTG molecules. Upon immobilization of tTG, obvious aggregation of the tTG biomolecules on the FMBs surface indicated successful immobilization of tTG. The BSA-attached FMBs (Fig. 4C) had a rough surface feature, and increases were seen in their size. The FMBs in Fig. 4D had a different surface morphology, indicating that Fe_3_O_4_@SiO_2_@3-PPA@tTG@BSA was coated by anti-tTG antibody molecules.

### Optimization of magneto immunosensor conditions

**Fig. 5:**
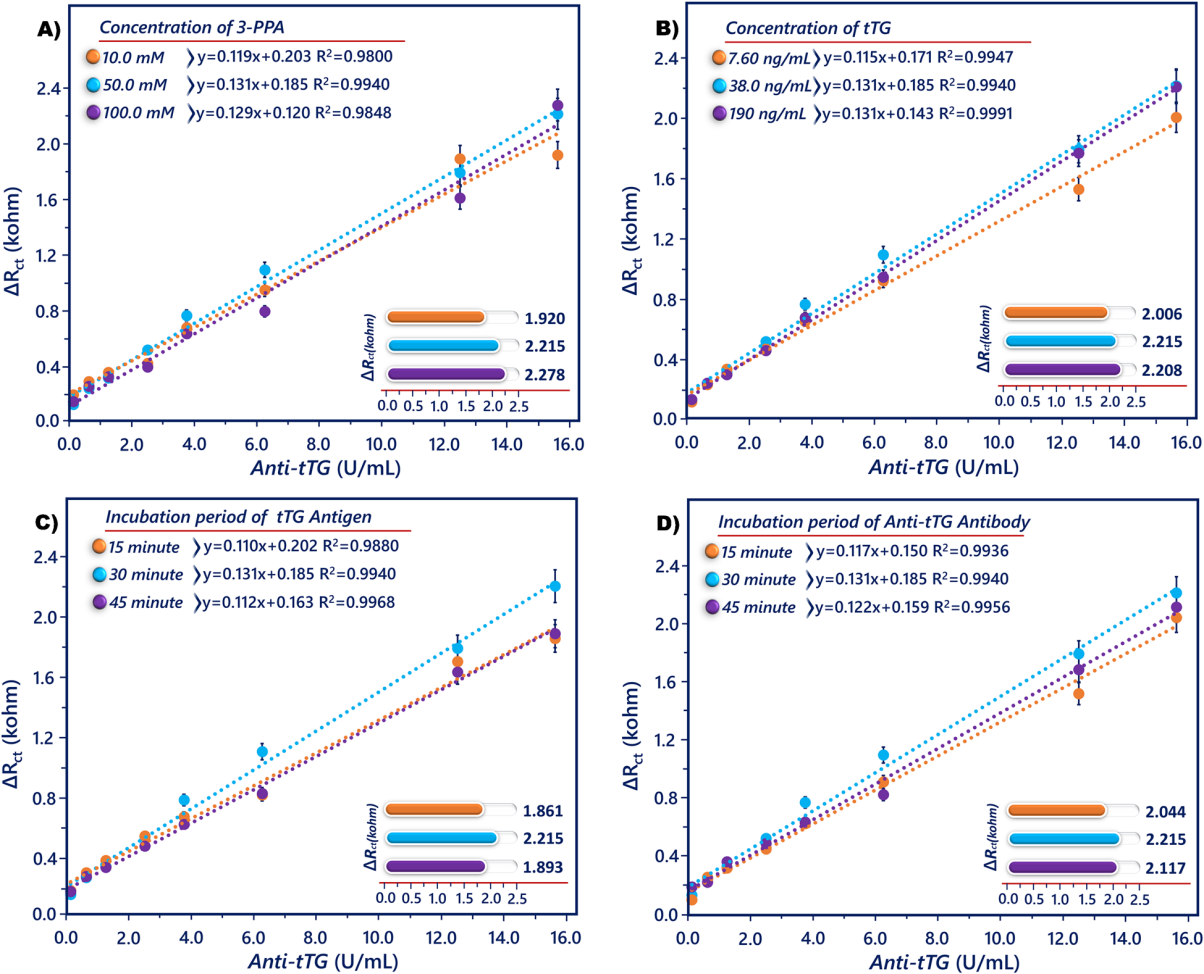
Effect of 3-PPA (**A**) and tTG (**B**) concentration on the EIS response, and the effect of tTG (**C**) and anti-tTG incubation time on the EIS response of the immunosensor (∆*R*_ct_ values obtained during optimization studies, inset)

Factors that could influence a response between the tTG antigen and anti-tTG antibody include 3-PPA and tTG concentration, and incubation time of biomolecules should be optimized. During the optimization studies, only one variable was changed, and the other variables were kept constant. The proposed magneto biosensor responses were monitored by the EIS technique, and the highest measured magneto biosensor signals (∆*R*_ct_) are given in the Fig. 5 inset. Firstly, the 3-PPA amount utilized for the FMBs construction was optimized. The immobilization of the biorecognition molecules may be sterically hampered by a high density of carboxylic acid groups, whereas the immunosensor’s sensitivity will be reduced by a low density of reaction groups. To investigate the 3-PPA concentration dependency of the immunosensor responses toward anti-tTG, the Fe_3_O_4_@SiO_2_ nanoparticles were incubated in 3-PPA solution at various concentrations ranging from 10 to 100 mM (38 ng/mL tTG concentration, 30 min tTG incubation time, and 30 min anti-tTG incubation time). As observed in Fig. 5A, the immunosensor illustrated high EIS responses at 50 and 100 mM. So, 50 mM was chosen as the best 3-PPA concentration because the signals that were obtained after using 50 and 100 mM 3-PPA were similar.

The concentration of tTG antigen was correlated with the immunosensor’s performance as determined by the immunoaffinity reaction. The EIS response rose as the tTG concentration rose, reaching its maximum value at 38 ng/mL, as seen in Fig. 5B (50 mM 3-PPA concentration, 30 min tTG incubation time, and 30 min anti-tTG incubation time). Thus, 38 ng/mL was chosen as the ideal tTG concentration. The incubation time is one of the important parameters for both immobilizing tTG and capturing anti-tTG antibody. The impact of the tTG molecule’s incubation period on the immunosensor signal was investigated (50 mM concentration, 38 ng/mL tTG concentration, and 30 min anti-tTG incubation time). The EIS response rose quickly as the incubation duration was increased from 15 to 30 min, as Fig. 5C illustrates. At the end of 15 min stirring, a low magneto biosensor response was obtained because not enough tTG was bound to the surface of the FMBs. The EIS response was not enhanced by longer incubation times. In fact, a decrease in magneto biosensor response was observed after 45 min stirring, which may be due to protein denaturation. Consequently, the ideal incubation period for tTG immobilization was determined to be 30 min. The immune response between tTG and anti-tTG was related to incubation duration. The BSA-modified FMBs were incubated for different times (15, 30, and 45 min) in an anti-tTG solution (50 mM concentration, 38 ng/mL tTG concentration, and 30 min tTG incubation time). The EIS response rose when the incubation duration was extended from 15 to 30 min, as seen in Fig. 5D, and the EIS signal tended to a steady value after 30 min. Longer duration did not lead to an increase in biosensor response. Thus, the ideal incubation duration for anti-tTG was determined to be 30 min.

### Biosensing performance of the anti-tTG immunosensor

**Fig. 6:**
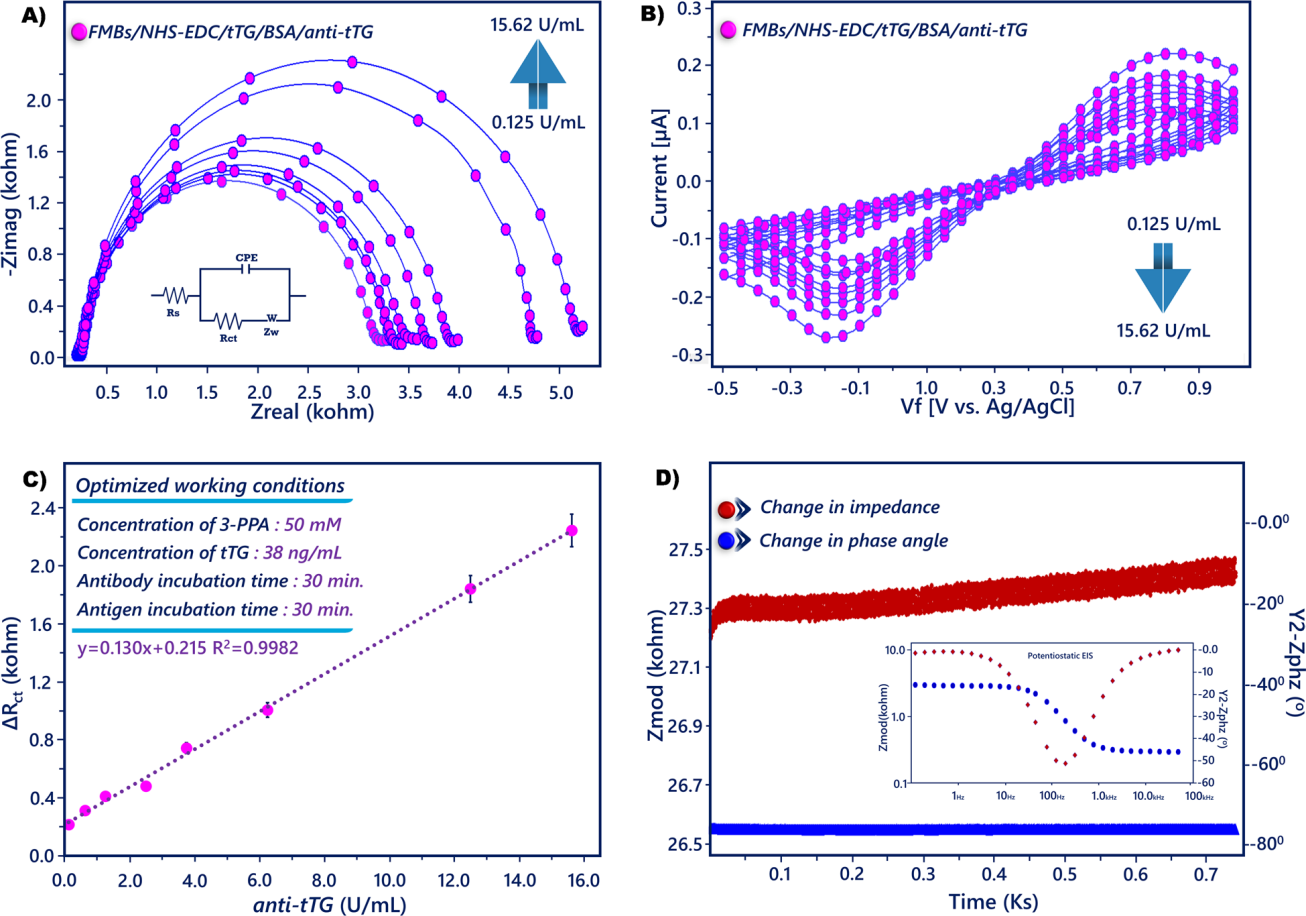
**A** EIS and **B** CV responses of the biosensor to different concentrations of anti-tTG. **C** Calibration plot. **D** SFI spectra of anti-tTG magneto biosensor and Bode plot (inset)

Under ideal experimental circumstances, 10 mL of ferri-ferro solution (5 mM, pH 7.0) was used to assess the immunosensor’s EIS and CV in relation to different anti-tTG concentrations. Figure 6A and B shows that the EIS response gradually increased, and CV peak current gradually declined with increasing amounts of anti-tTG because of the formation of an insulating layer of protein molecules.

The EIS responses were analyzed as a function of the anti-tTG amount. In particular, using tTG as the biorecognition molecule, the amount of anti-tTG in the range from 0.125 to 15.62 U/mL was directly correlated with the increased EIS signal (Fig. 6C). Since the hydrophobic protein layer of the immunocomplex would prevent the electrochemical probe’s electron transfer, the semicircle width increased proportionately with the anti-tTG concentration, indicating that the electrode interface had captured the anti-tTG. The regression equation was Δ*R*_ct_ (U/mL) = 0.130[anti-tTG] + 0.215, *R*^2^ = 0.9981, and the LOD of anti-tTG was 0.04 U/mL. Apart from EIS and CV measurements, the single frequency impedance (SFI) technique was utilized to track the variations in electrochemical impedance throughout time. In addition, simultaneous examination of variations on the electrode surface is important, and this technique provides information about the kinetic binding between antibody and antigen. This method used the Bode plot to determine the constant frequency at which electrochemical impedance was periodically recorded. The real-time reaction between the tTG and anti-tTG present on the magnetic nanoparticles was studied in PBS (50 mM, pH 7.0) containing anti-tTG. A frequency of 45 Hz was utilized for the SFI analysis. As time passed, the SFI plot, which is depicted in Fig. 6D, revealed an increase in antigen–antibody interaction.

The immunosensor’s performance was contrasted with that of ELISA kits and other documented biosensors. Compared with other anti-tTG analysis techniques, the suggested immunosensor had an excellent performance in the LOD and the dynamic range (Table 1). For instance, the LOD was lower than that of analysis methods such as the electrochemical immunosensor based on biotin-quantum dots (QDs)-streptavidin (3 U/mL) [39] and multiwalled carbon nanotubes (MWCNTs)-gold nanoparticles (AuNPs) nanocomposite (2.45 U/mL) [40] (Table 1). These findings adequately demonstrated the effectiveness of using 3-PPA-coated magnetic nanoparticles for magnetosensing.

**Table 1:** Comparison of the magneto biosensor and other anti-tTG analysis techniques

Analysis technique	Linear range (U/mL)	Detection limit (U/mL)	Reference
QDs modified 8-channel immunosensor	3–100	3	[39]
MWCNTs and AuNPs modified immunosensor	0–40	-	[41]
MWCNTs and AuNPs modified dual immunosensor	0–100	2.45	[40]
DNA oligomer attached biosensor	0.01–10	0.01	[42]
ELISA	0–200	10	Orgentec
ELISA	0–100	10	MyBiosource
FMBs based magneto biosensor	0.125–15.62	0.04	This study

**Fig. 7:**
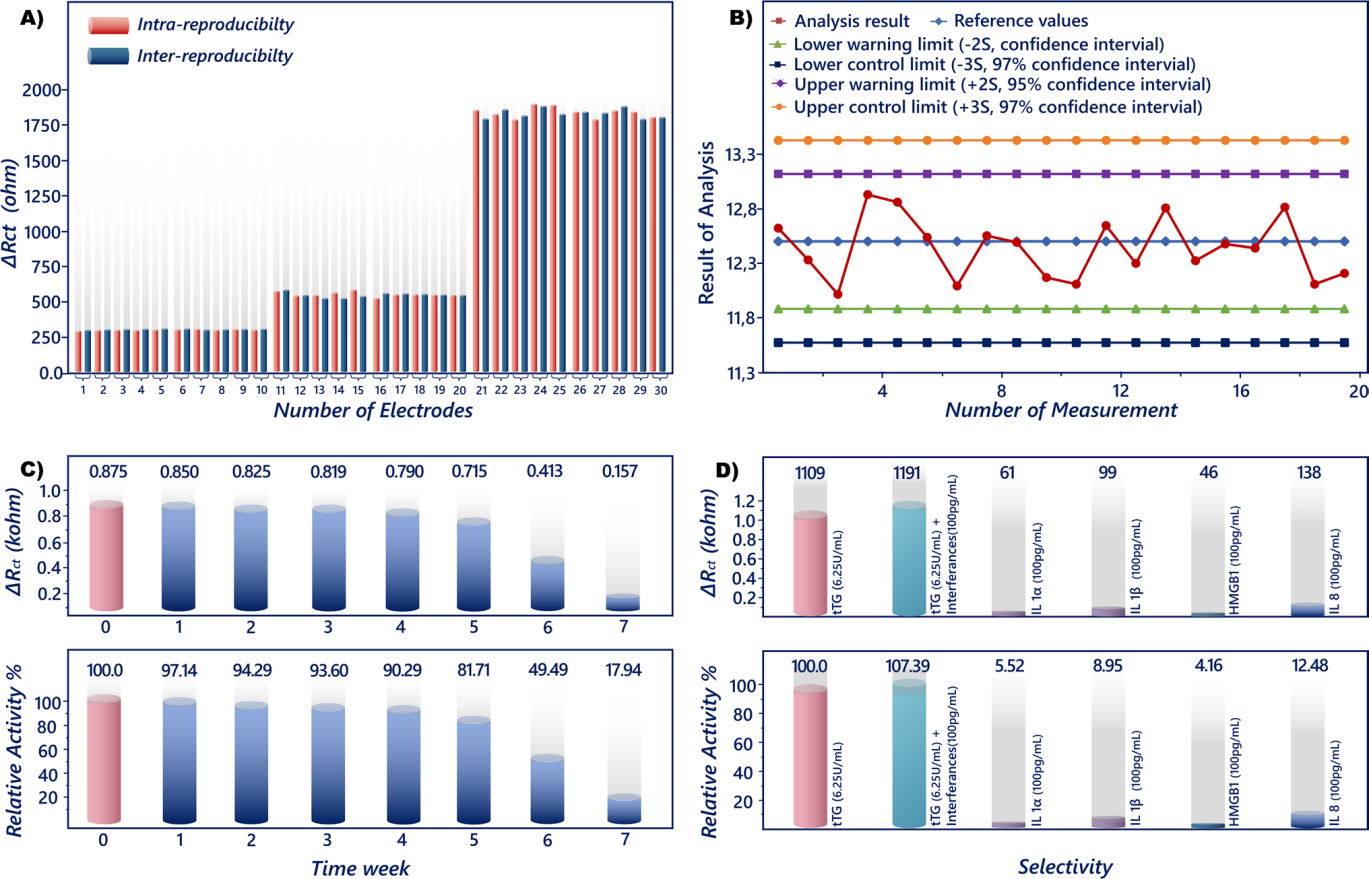
Results of the biosensor intra- and inter-reproducibility studies (**A**), quality of control plot of the biosensor (**B**), results of selectivity (**C**), and storage stability (**D**) studies

Intra- and inter-reproducibility are important properties of the electrochemical biosensors, which illustrate the performance of the biosensing system [43]. The reproducibility of the proposed biosensor was assessed using impedimetric intra- and inter-day measurements (Fig. 7A). For this reason, BSA-blocked FMBs were utilized to measure three different anti-tTG concentrations (0.625, 2.5, and 12.5 U/mL). To evaluate intra-assay measurements, these three different anti-tTG concentrations were measured 10 times on the same day. The coefficients of variation (CVs) calculated for intra-day reproducibility were found to be 4.74, 5.07, and 2.12% for 0.625, 2.5, and 12.5 U/mL anti-tTG, respectively. To evaluate inter-day measurements, these three different anti-tTG concentrations were measured 10 times on different days. The CVs calculated for inter-assay 2.12, 5.04, and 2.48% for 0.625, 2.5, and 12.5 U/mL anti-tTG, respectively. According to the experimental findings, the recommended biosensor’s reproducibility was deemed satisfactory. Low CVs were also noted for the magneto biosensor when compared to biosensors that utilized commercial magnetic nanoparticles. In addition, the intra- and inter-day measurement results were analyzed over time with a quality control chart, and upper and lower control limits and upper and lower warning limits were calculated by using the + / − 2 s and + / − 3 s, respectively. The average value of the chart was 12.5 U/mL. The upper and lower control limits and upper and lower warning limits were 13.43 and 11.57 and 13.12 and 11.88, respectively. This result illustrated that the analysis results varied within the control limits (Fig. 7B).

In diagnostic applications, interference-induced nonspecific signals have a significant impact on the sensor response [44]. In this study, the immunosensor’s selectivity was evaluated in different biomarker solutions, including IL1α (100 pg/mL), IL1β (100 pg/mL), IL8 (100 pg/mL), HMGB1 (100 pg/mL), and anti-tTG (6.25 U/mL). For this purpose, Fe_3_O_4_@SiO_2_@3-PPA@tTG@BSA magnetic beads were stirred for 30 min in these biomarkers’ solution (20 µL), and after stirring, these nanoparticles were separated and washed with MES buffer (50 mM, pH 6) to get rid of any physically bound biomolecules. After that, the electrochemical signals of these nanoparticles were recorded. The immunosensor displayed a strong signal to the anti-tTG antibody and virtually minimal reaction to nonspecific proteins, as seen in Fig. 7C. This impedimetric result demonstrated that the anti-tTG immunosensor’s selectivity was adequate. The excellent selectivity may have originated from the successful fabrication of Fe_3_O_4_@SiO_2_@3-PPA@tTG and the high bioaffinity between tTG and the anti-tTG antibody.

Determining an immunosensor’s storage stability is crucial for proving the biorecognition component’s activity and immobilization effectiveness [45].

In order to test the storage stability, Fe_3_O_4_@SiO_2_@3-PPA@tTG@BSA magnetic beads were stored at 4 °C. Each week, these particles were stirred for 30 min in anti-tTG solution (5 U/mL, 20 µL), and after stirring, these nanoparticles were separated and washed with MES buffer (50 mM, pH 6) to get rid of any physically bound biomolecules. After that, the electrochemical signals of these nanoparticles were recorded. The immunosensor could retain its initial reaction for seven days when kept at 4 °C, and after 21 days of storage, the impedimetric response was 93.60% of the initial response. As a result, the immunosensor was appropriate for clinical diagnostics and had a respectable storage stability. Even after a very long period of 35 days, it exhibited 81.71% of its activity (Fig. 7D). These results show that the proposed biosensor can be used for long periods of time without experiencing a noticeable decline in activity. Additionally, the suggested magneto biosensor showed satisfactory stability and a lengthy storage period when compared to other published studies.

### Application in clinical samples

**Fig. 8:**
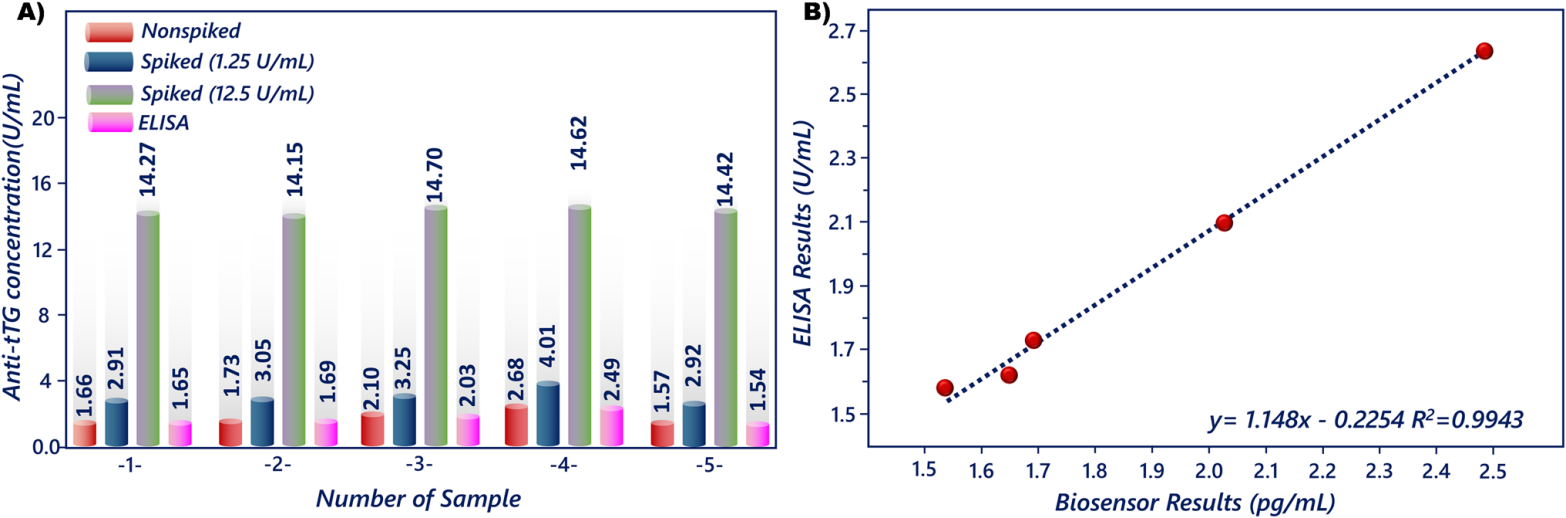
Results obtained by the suggested magneto biosensor and ELISA (**A**) and correlation between magneto biosensor and ELISA measurements (**B**)

The accuracy and practical viability of the magneto sensor for analysis of anti-tTG in biological samples were also assessed. Therefore, five serum samples were employed after fivefold dilution, and the standard addition method was utilized to analyze the applicability of the magneto biosensor. Recovery test samples were prepared by adding different amounts (1.25 and 12.5 U/mL) of anti-tTG to serum samples. The result of each sample was determined two times and summarized in Table 2, which showed the recoveries in the range of 96.56–103.47% for serum samples, indicating that the magneto biosensing approach was reliable. Furthermore, the amount of anti-tTG in serum samples was also measured by ELISA in addition to the magneto biosensor, and the analysis results were compared with ELISA as the reference method. The result of the comparison is shown in Fig. 8A.

**Table 2:** Recovery test result for serum samples

Serum sample (U/mL)	Added(U/mL)	Detected (U/mL)	RSD (*n* = 2, %)	Recovery (%)
1.70/1.62	1.25	2.92/2.90	0.56	99.93
	12.5	14.68/13.87	4.01	100.79
1.72/1.74	1.25	2.99/3.10	2.68	102.24
	12.5	14.29/14.01	1.42	99.45
2.12/2.07	1.25	3.22/3.29	1.51	97.29
	12.5	14.96/14.43	2.56	100.7
2.69/2.59	1.25	4.07/3.95	2.04	103.10
	12.5	14.22/15.02	3.87	96.56
1.55/1.59	1.25	2.90/2.93	0.56	103.47
	12.5	14.54/14.31	1.13	102.53

Comparisons were made using linear regression analysis, and the assay variability was measured by the resulting correlation coefficients, whereas assay responsiveness variations were shown by the correlations’ slopes. The slope of the correlation of serum sample analysis was 1.03, illustrating that the biosensor’s quantitative results were marginally higher than those derived from ELISA analysis (Fig. 8B). This finding showed that the magneto biosensor was sensitive to anti-tTG measurements.

Moreover, the obtained data (Table 2) illustrated that the developed biosensor was suitable for measuring the concentration of anti-tTG in serum samples. In fact, the developed system was more sensitive than the ELISA kit and was more successful than the ELISA kit in measuring anti-tTG levels in healthy individuals. In addition, in this study, the serum samples were analyzed at a fivefold dilution; in this case, this biosensor could measure anti-tTG up to a concentration of 78.1 U/mL. If the anti-tTG concentration is higher than this value, further dilution can be performed. Biosensors with a wider linear range can be produced with the use of nanomaterials and polymer during the modification of magnetic beads.

## Conclusion

In this study, a type of simple and ultrasensitive impedimetric magneto biosensor to detect anti-tTG antibody concentration was developed successfully. The FMBs were employed as a biorecognition element immobilization matrix material, and a low-cost ITO substrate was utilized as a fixed support platform for electrochemical sensing. The structure of 3-PPA allowed the resulting FMBs to have a lot of anchoring points, which provided immobilization of tTG proteins. The detection mechanism of the proposed biosensor was based on the immunochemical reaction between tTG and anti-tTG due to the high affinity between these biomolecules. Electrochemical methods such as EIS, CV, DPV, and SWV were used to describe the tTG–anti-tTG binding interaction at the electrode–electrolyte interface in order to detect anti-tTG with sensitivity and selectivity. Additionally, the electrode surface of the magneto biosensor was characterized by SEM and SEM–EDS to support the uniform and stable response suitable for biosensing. Under the ideal circumstances, the suggested impedimetric magneto sensor demonstrated outstanding performance for the determination of anti-tTG with a wide linear range from 0.125 to 15.62 U/mL and a low LOD of 0.04 U/mL. The biocompatible microenvironment of the biomolecules and the huge specific surface area of the FMBs substrate may be responsible for the immunosensor’s exceptional performance. The results also displayed that the suggested impedimetric magneto biosensor had acceptable stability, excellent specificity, and good reproducibility. Finally, the practical applicability of the magneto biosensor was verified by analyzing real samples, and the anti-tTG levels were compared with ELISA anti-tTG analysis results. A correlation between anti-tTG serum levels was found by a standard ELISA and those obtained with the FMBs-based magneto biosensor. Consequently, it was anticipated to offer a workable platform for the analysis of biomarkers in the early diagnosis and management of CsD.

## Supplementary Information

Below is the link to the electronic supplementary material.

ESM 1 (DOCX 18.2 MB)

## Data Availability

Data is provided within the manuscript or supplementary information files.

## References

[1] Habtamu HB, Sentic M, Silvestrini M, De Leo L, Not T, Arbault S et al (2015) A sensitive electrochemiluminescence immunosensor for celiac disease diagnosis based on nanoelectrode ensembles. Anal Chem 87(24):12080–12087. 10.1021/acs.analchem.5b02801 26556023 10.1021/acs.analchem.5b02801

[2] Rajput MS, Chauhan A, Makharia GK. Epidemiology and clinical features of celiac disease in adults. (2022) Coeliac Disease and Gluten-Related Disorders: Elsevier; p. 1–23. 10.1016/B978-0-12-821571-5.00012-X

[3] Hedesh M, Maharat Z, Khalaji A, Pazouki L, Dooraki S. (2024) Early detection of celiac disease through its common symptoms using machine learning algorithms. Journal of Clinical Images and Medical Case Reports. 5(3):2915. 10.52768/2766-7820/2915

[4] Jain S, Lamba BY, Dubey SK (2024) Recent advancements in the sensors for food analysis to detect gluten: a mini-review [2019–2023]. Food Chem 139204:139204. 10.1016/j.foodchem.2024.139204 10.1016/j.foodchem.2024.139204 38613992

[5] Walker MM, Murray JA (2011) An update in the diagnosis of coeliac disease. Histopathology 59(2):166–179. 10.1111/j.1365-2559.2010.03680.x 21054494 10.1111/j.1365-2559.2010.03680.x

[6] Pasinszki T, Krebsz M (2018) Biosensors for non-invasive detection of celiac disease biomarkers in body fluids. Biosensors 8(2):55. 10.3390/bios8020055 29914179 10.3390/bios8020055 PMC6023018

[7] Dulay S, Lozano-Sánchez P, Iwuoha E, Katakis I, O’Sullivan CK (2011) Electrochemical detection of celiac disease-related anti-tissue transglutaminase antibodies using thiol based surface chemistry. Biosens Bioelectron 26(9):3852–3856. 10.1016/j.bios.2011.02.045 21420846 10.1016/j.bios.2011.02.045

[8] Vives-Pi M; Takasawa S, Pujol-Autonell I, Planas R, Cabre E, Ojanguren I, Montraveta M, et al. (2013) Biomarkers for diagnosis and monitoring of celiac disease. Journal of clinical gastroenterology. J. Clin. Gastroenterol. 47(4):p 308–313. 10.1097/MCG.0b013e31827874e3 10.1097/MCG.0b013e31827874e3 23388848

[9] Pividori M, Lermo A, Bonanni A, Alegret S, Del Valle M (2009) Electrochemical immunosensor for the diagnosis of celiac disease. Anal Biochem 388(2):229–234. 10.1016/j.ab.2009.02.026 19250919 10.1016/j.ab.2009.02.026

[10] Engin B, Huseynova C, Ak T, Ayla A.Y, Can G, Uğurlu S. (2023). Screening of antigliadin and antitissue transglutaminase antibodies in patients with chronic plaque psoriasis: a case-control study. Turk. J. Med. Sci. 53: 544–551. 10.55730/1300-0144.5615 10.55730/1300-0144.5615 PMC10387849 37476878

[11] Kamilova AT, Gulnoza KA, Zulkhumar EU, Dilroba AA, Swetlana IG (2022) The activity of antimicrobial peptides in pediatric celiac disease. Front Pediatr 10:873793. 10.3389/fped.2022.873793 35733815 10.3389/fped.2022.873793 PMC9208658

[12] Ajdani M, Mortazavi N, Besharat S, Mohammadi S, Amiriani T, Sohrabi A, Norouzi A, Edris G (2022) Serum and salivary tissue transglutaminase IGA (tTG-IGA) level in celiac patients. BMC Gastroenterol 22(1):375. 10.1186/s12876-022-02456-x 35933327 10.1186/s12876-022-02456-x PMC9357310

[13] Nassef HM, Bermudo Redondo MC, Ciclitira PJ, Ellis HJ, Fragoso A, O’Sullivan CK (2008) Electrochemical immunosensor for detection of celiac disease toxic gliadin in foodstuff. Anal Chem 80(23):9265–9271. 10.1021/ac801620j 19551990 10.1021/ac801620j

[14] Shamsazar A, Asadi A, Seifzadeh D, Mahdavi M. (2021) A novel and highly sensitive sandwich-type immunosensor for prostate-specific antigen detection based on MWCNTs-Fe_3_O_4_ nanocomposite. Sens. Actuators, B. 346:130459. 10.1016/j.snb.2021.130459

[15] Aydın EB, Aydın M, Sezgintürk MK (2017) A highly sensitive immunosensor based on ITO thin films covered by a new semi-conductive conjugated polymer for the determination of TNFα in human saliva and serum samples. Biosens Bioelectron 97:169–176. 10.1016/j.bios.2017.05.056 28599176 10.1016/j.bios.2017.05.056

[16] Dutta G et al (2014) Washing-free heterogeneous immunosensor using proximity-dependent electron mediation between an enzyme label and an electrode. Anal Chem 86(9):4589–4595 24758236 10.1021/ac5006487

[17] Gupta S, Kaushal A, Kumar A, Kumar D (2019) Recent advances in biosensors for diagnosis of celiac disease: a review. Biotechnol Bioeng 116(2):444–451. 10.1002/bit.26856 30516838 10.1002/bit.26856

[18] Mukherjee B, Mandal M, Suresh RR, Kar S, Parida BK, Chakraborty S, Dutta G (2025) A non-enzymatic highly stable electrochemical sensing platform based on allylamine capped copper nanoparticles for the detection of the soil nitrate content. Analyst 150:936–952. 10.1039/D4AN01345J 39898593 10.1039/d4an01345j

[19] Dutta G, Nagarajan S, Lapidus LJ, Lillehoj PB (2017) Enzyme-free electrochemical immunosensor based on methylene blue and the electro-oxidation of hydrazine on Pt nanoparticles. Biosens Bioelectron 92:372–377. 10.1016/j.bios.2016.10.094 27829560 10.1016/j.bios.2016.10.094 PMC5342929

[20] Pasinszki T, Krebsz M (2019) Advances in celiac disease testing. Adv Clin Chem 91:1–29. 10.1016/bs.acc.2019.03.001 31331486 10.1016/bs.acc.2019.03.001

[21] Elshafey R, Tavares AC, Siaj M, Zourob M (2013) Electrochemical impedance immunosensor based on gold nanoparticles–protein G for the detection of cancer marker epidermal growth factor receptor in human plasma and brain tissue. Biosens Bioelectron 50:143–149. 10.1016/j.bios.2013.05.063 23850780 10.1016/j.bios.2013.05.063

[22] Aydın EB, Aydın M, Sezgintürk MK (2021) Fabrication of electrochemical immunosensor based on acid-substituted poly (pyrrole) polymer modified disposable ITO electrode for sensitive detection of CCR4 cancer biomarker in human serum. Talanta 222:121487. 10.1016/j.talanta.2020.121487 33167207 10.1016/j.talanta.2020.121487

[23] Aydın EB, Aydın M, Sezgintürk MK (2019) Ultrasensitive determination of cadherin-like protein 22 with a label-free electrochemical immunosensor using brush type poly (thiophene-g-glycidylmethacrylate) modified disposable ITO electrode. Talanta 200:387–397. 10.1016/j.talanta.2019.03.082 31036200 10.1016/j.talanta.2019.03.082

[24] Xiang W, Lv Q, Shi H, Xie B, Gao L (2020) Aptamer-based biosensor for detecting carcinoembryonic antigen. Talanta 214:120716. 10.1016/j.talanta.2020.120716 32278406 10.1016/j.talanta.2020.120716

[25] Nguyen MD, Tran H-V, Xu S, Lee TR (2021) Fe_3_O_4_ nanoparticles: structures, synthesis, magnetic properties, surface functionalization, and emerging applications. App Sci 11(23):11301. 10.3390/app112311301 10.3390/app112311301 PMC9285867 35844268

[26] Aydın M, Aydın EB, Sezgintürk MK. (2024) Label-free and ultrasensitive electrochemical cotinine sensing based on bio-modified magnetic nanoparticles. Sens. Actuators, B. 408:135476. 10.1016/j.snb.2024.135476

[27] Sarkar TN, Dutta DG (2023) A new biosensing platform based on L-cysteine-capped Fe3O4 nanoparticles embedded in chitosan-MWCNT matrix: electrochemical kinetic and sensing studies. Biosens Bioelectron 15:100412. 10.1016/j.biosx.2023.100412

[28] Chauhan N, Pundir CS (2012) An amperometric acetylcholinesterase sensor based on Fe_3_O_4_ nanoparticle/multi-walled carbon nanotube-modified ITO-coated glass plate for the detection of pesticides. Electrochim Acta 67:79–86. 10.1016/j.electacta.2012.02.012

[29] Ma Y, Zheng B, Zhao Y, Yuan H, Cai Y, Du J, Xiao D (2013) A sensitive and selective chemosensor for GSSG detection based on the recovered fluorescence of NDPA-Fe_3_O_4_@ SiO_2_-Cu (II) nanomaterial. Biosens Bioelectron 48:138–144. 10.1016/j.bios.2013.04.006 23669046 10.1016/j.bios.2013.04.006

[30] Peng X, Wang Y, Tang X, Liu W (2011) Functionalized magnetic core–shell Fe_3_O_4_@SiO_2_ nanoparticles as selectivity-enhanced chemosensor for Hg (II). Dyes Pigm 91(1):26–32. 10.1016/j.dyepig.2011.01.012

[31] Aydın M, Aydın EB, Sezginturk MK (2024) Carboxyethylsilanetriol-coated magnetic nanoparticles as an ultrasensitive immunoplatform for electrochemical magnetosensing of cotinine. ACS Biomater Sci Eng 10(4):2567–2580. 10.1021/acsbiomaterials.3c01872 38529538 10.1021/acsbiomaterials.3c01872

[32] Cao H, He J, Deng L, Gao X (2009) Fabrication of cyclodextrin-functionalized superparamagnetic Fe3O4/amino-silane core–shell nanoparticles via layer-by-layer method. Appl Surf Sci 255(18):7974–7980. 10.1016/j.apsusc.2009.04.199

[33] Aydın EB, Sezgintürk MK (2021) Ultrasensitive detection of interleukin 1α using 3-phosphonopropionic acid modified FTO surface as an effective platform for disposable biosensor fabrication. Bioelectrochemistry 138:107698. 10.1016/j.bioelechem.2020.107698 33254051 10.1016/j.bioelechem.2020.107698

[34] Wang L, Sun Y, Wang J, Wang J, Yu A, Zhang H, Song D. (2011) Preparation of surface plasmon resonance biosensor based on magnetic core/shell Fe_3_O_4_/SiO_2_ and Fe_3_O_4_/Ag/SiO_2_ nanoparticles. Colloids Surf., B. 84(2) 484–90. 10.1016/j.colsurfb.2011.02.003 10.1016/j.colsurfb.2011.02.003 21353500

[35] Aydın M, Aydın EB, Sezgintürk MK (2024) Functionalized magnetic nanoparticles for electrochemical magneto biosensing of PSMA cancer biomarker. New J Chem 48(13):5769–5781. 10.1039/D4NJ00274A

[36] Lv S, Sheng J, Zhao S, Liu M, Chen L (2018) The detection of brucellosis antibody in whole serum based on the low-fouling electrochemical immunosensor fabricated with magnetic Fe3O4@ Au@ PEG@ HA nanoparticles. Biosens Bioelectron 117:138–144. 10.1016/j.bios.2018.06.010 29894850 10.1016/j.bios.2018.06.010

[37] Zhuo Y, Yuan P-X, Yuan R, Chai Y-Q, Hong C-L (2009) Bienzyme functionalized three-layer composite magnetic nanoparticles for electrochemical immunosensors. Biomaterials 30(12):2284–2290. 10.1016/j.biomaterials.2009.01.002 19162316 10.1016/j.biomaterials.2009.01.002

[38] de Sousa JR, Parente MMV, Diógenes ICN, Lopes LGF, de Lima NP, Temperini MLA et al (2004) A correlation study between the conformation of the 1, 4-dithiane SAM on gold and its performance to assess the heterogeneous electron-transfer reactions. J Electroanal Chem 566(2):443–449. 10.1016/j.jelechem.2003.12.010

[39] Martin-Yerga D, Costa-Garcia A (2015) Towards a blocking-free electrochemical immunosensing strategy for anti-transglutaminase antibodies using screen-printed electrodes. Bioelectrochemistry 105:88–94. 10.1016/j.bioelechem.2015.05.014 26043306 10.1016/j.bioelechem.2015.05.014

[40] Neves MM, González-García MB, Delerue-Matos C, Costa-García A (2013) Multiplexed electrochemical immunosensor for detection of celiac disease serological markers. Sens Actuators, B 187:33–39. 10.1016/j.snb.2012.09.019

[41] Neves MM, González-García MB, Nouws HP, Costa-García A (2012) Celiac disease detection using a transglutaminase electrochemical immunosensor fabricated on nanohybrid screen-printed carbon electrodes. Biosens Bioelectron 31(1):95–100. 10.1016/j.bios.2011.09.044 22019096 10.1016/j.bios.2011.09.044

[42] Nguyen AB, Maldonado M, Poch D, Sodia T, Smith A, Rowland TJ, Bonham AJ (2021) Electrochemical DNA biosensor that detects early celiac disease autoantibodies. Sensors 21(8):2671. 10.3390/s21082671 33920183 10.3390/s21082671 PMC8070315

[43] Atta NF, Galal A, El-Gohary AR (2022) Electrochemical sensing of dobutamine, paracetamol, amlodipine, and daclatasvir in serum based on thiourea SAMs over nano-gold particles–CNTs composite. New J Chem 46(25):12265–12277. 10.1039/D2NJ01822E

[44] Zhang C, Shen G, Shen Y, Zhang X (2015) The development of an electrochemical immunosensor using a thiol aromatic aldehyde and PAMAM-functionalized Fe_3_O_4_@ Au nanoparticles. Anal Biochem 485:66–71. 10.1016/j.ab.2015.06.016 26087149 10.1016/j.ab.2015.06.016

[45] Wu J, Tang J, Dai Z, Yan F, Ju H, El Murr N (2006) A disposable electrochemical immunosensor for flow injection immunoassay of carcinoembryonic antigen. Biosens Bioelectron 22(1):102–108. 10.1016/j.bios.2005.12.008 16427775 10.1016/j.bios.2005.12.008

