# Supplementary material for: Early and sensitive diagnosis of celiac autoimmune disease by using carboxylic acid functionalized magnetic nanoparticles-assisted biosensing platform
Source: Mikrochim Acta. 2025 Apr 8;192(5):284. doi: 10.1007/s00604-025-07129-6 (PMC11978692; doi:10.1007/s00604-025-07129-6)
Supplement: Supplementary file 1 — (DOCX 18.2 MB) [file 604_2025_7129_MOESM1_ESM.docx]

Supplementary Information for

**Early and sensitive diagnosis of celiac autoimmune disease by using carboxylic acid functionalized magnetic nanoparticles assisted biosensing platform**

**Elif Burcu Aydın^a*^, Muhammet Aydın^a^, Mustafa Kemal Sezgintürk^b^**

**^a^Tekirdağ Namık Kemal University, Scientific and Technological Research Center, Tekirdağ-TURKEY**

**^b^Çanakkale Onsekiz Mart University, Faculty of Engineering, Bioengineering Department, Çanakkale-TURKEY**

***Corresponding Author:** Assoc. Prof. Elif Burcu Aydın

**e-mail:** [ebbahadir@nku.edu.tr](mailto:ebbahadir@nku.edu.tr)

**Chemical Synthesis and Characterization Details**

Fig. SI-1 presents FTIR spectra of Fe_3_O_4_, Fe_3_O_4_@SiO_2_ and Fe_3_O_4_@SiO_2_@3-PPA (FMBs) nanoparticles. The peaks at 547 and 437 cm^−1^ illustrated the internal tension peaks of magnetic nanoparticles (Fig. SI-1A). The Si-O-Si asymmetric and symmetric stretching vibration bands appeared at 1058 and 807 cm^−1^, respectively, which confirmed the exis­tence of TEOS coating on magnetic nanoparticles (Fig. SI-1B) [1, 2]. These results indicate successful synthesis of Fe_3_O_4_@SiO_2_ nanoparticles. In addition, the band between 3000−3500 cm^−1^ was attributed to stretching vibration of the Si−OH. Fig. SI-1C shows the FTIR spectrum of FMBs. It can be seen that the band at 1705 cm^−1^ was associated with the stretching vibration of C=O bond of carboxyl groups on 3-PPA, which proved the existence of 3-PPA on functionalized magnetic nanoparticles.

| 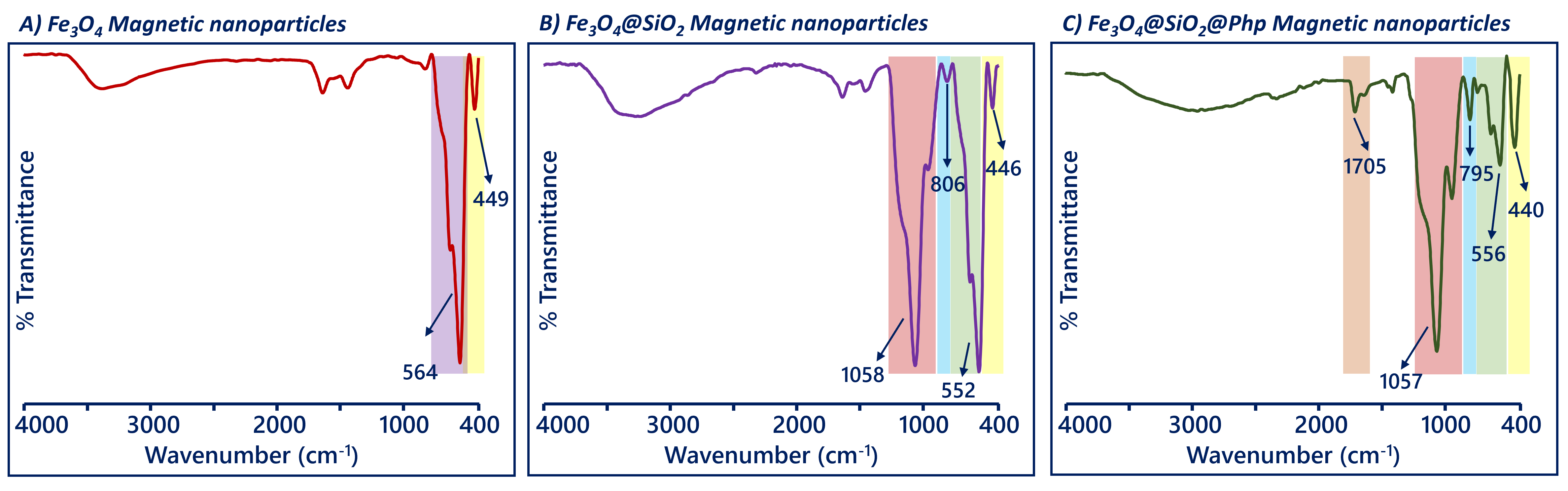 |
| --- |
| **Figure SI-1.** FTIR spectra of Fe_3_O_4_, Fe_3_O_4_@SiO_2_ and Fe_3_O_4_@SiO_2_@3-PPA magnetic beads. |

The Raman spectra of Fe_3_O_4_, Fe_3_O_4_@SiO_2_, and Fe_3_O_4_@SiO_2_@3-PPA magnetic nanoparticles are illustrated in Fig. SI-2. As a result of Raman analysis, the phase of the functionalized magnetic beads was determined [3]. The characteristic T_1g_ and A_1g_ peaks appeared at (361, 494, and 678 cm^−1^), (356, 506, and 692 cm^−1^), and (346, 511, and 705 cm^−1^) for Fe_3_O_4_, Fe_3_O_4_@SiO_2_, and FMBs, respectively. This A_1g_ peak indicated the magnetite phase of FMBs. The little differences were seen in the peaks of A1g and T_1g_, illustrating the formation of Fe_3_O_4_@SiO_2_ and FMBs.

| 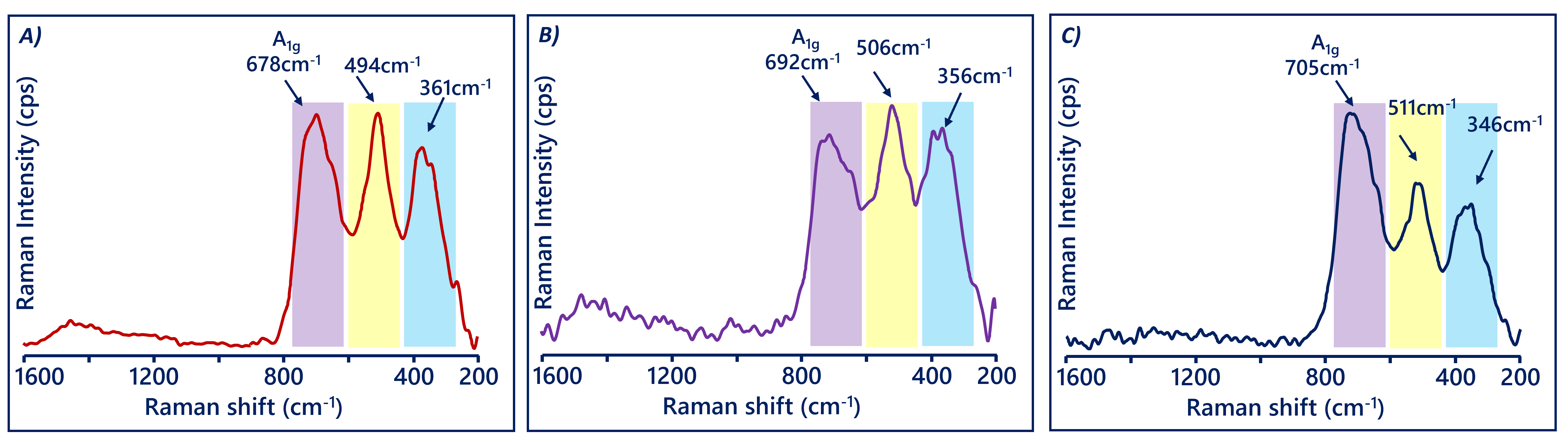 |
| --- |
| **Figure SI-2.** Raman spectra of Fe_3_O_4_, Fe_3_O_4_@SiO_2_, and Fe_3_O_4_@SiO_2_@3-PPA functionalized magnetic nanoparticles. |

The XRD pattern was further utilized to confirm the formation of Fe_3_O_4_ nanoparticles (Fig. SI-3). Six characteristic peaks at 2θ values of 30.6◦, 35.7◦, 43.3◦, 53.7◦, 57.4◦, and 63.3◦ were indexed to (220), (311), (400), (422), (511), and (440) crystal planes of Fe_3_O_4_ nanoparticles. These peaks were consistent with the database in JCPDS file no. 19-0629 and revealed that the resultant nanoparticles were pure Fe_3_O_4_ FMBs. The absence of other sharp and contamination-related peaks in the spectrum indicates that Fe_3_O_4_ particles were successfully synthesized by co-precipitation method [4].

| 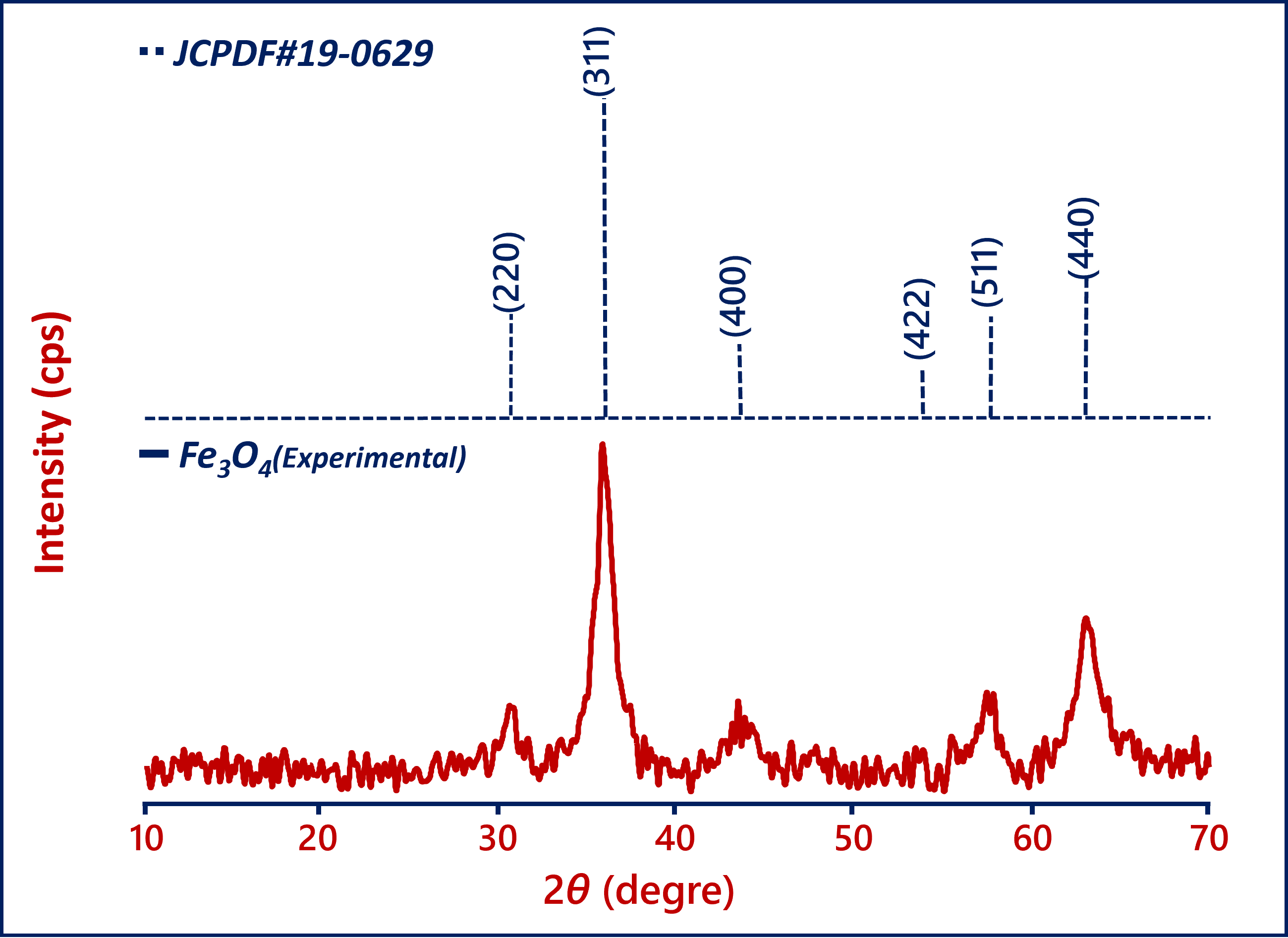 |
| --- |
| **Figure SI-3.** XRD pattern of FMBs. |

The SEM images provide more detailed morphological information on the Fe_3_O_4_, Fe_3_O_4_@SiO_2_, and FMBs. SEM analysis at different magnifications of magnetic nanoparticles illustrated that they were uniformly spherical structures (Fig.SI-4). In addition, the unique magnetic characteristics of pure Fe_3_O_4_ nanoparticles caused them to agglomerate. Upon deposition of SiO_2_ onto the Fe_3_O_4_ nanoparticles, the diameter of the particles increased by about 150 nm, demonstrating that the successful coating of SiO_2_ layer. The SiO_2_ layer-coated magnetic beads were spherical in structure and dispersed well with no signs of aggregation (Fig. SI-4B). After modification with 3-PPA, the diameter of FMBs became larger. As presented in Fig. SI-4C, it can be seen that the FMBs were well formed and highly dispersed with the size about 200 nm.

| 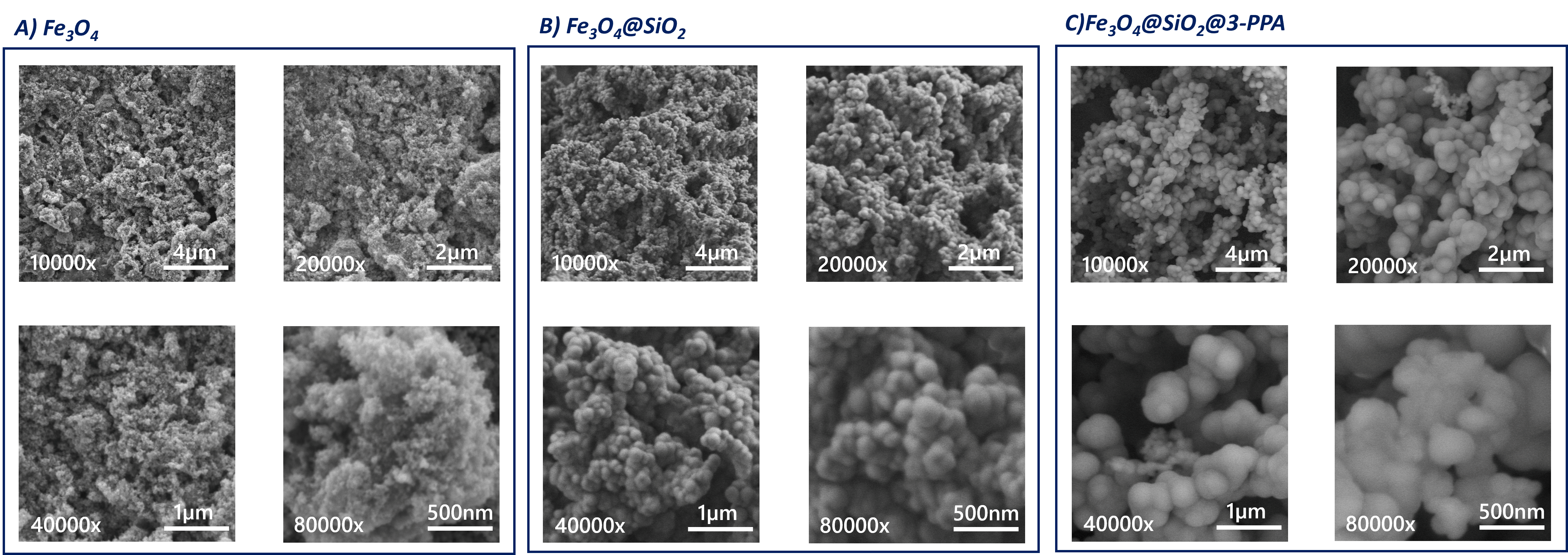 |
| --- |
| **Figure SI-4.** SEM images of Fe_3_O_4_, Fe_3_O_4_@SiO_2_, and Fe_3_O_4_@SiO_2_@3-PPA magnetic nanoparticles. |

The SEM-EDS analysis was used to investigate the successful construction of the FMBs. The EDS data (Fig. SI-5) of pure Fe_3_O_4_ magnetic nanoparticles showed the existence of Fe and O elements in the sample and the magnetite phase of Fe_3_O_4_ nanoparticle. In addition, the mapping analysis displayed the homogeneous distribution of Fe and O elements in the sample. The binding energies of Fe were present at about 0.5, 6.4, and 7.1 keV, and this result was consistent with the literature. Following the deposition of SiO_2_ on Fe_3_O_4_ nanoparticles, EDS data showed that Fe, C, Si, and O elements were homogeneously distributed in the sample. The EDS spectrum of FMBs had P, Fe, C, Si and O elements, and P content increased due to 3-PPA coating, but Fe content decreased from 17 to 0.4 due to this coating. Thus, EDS analysis proved the fact of the successful construction of magnetic nanoparticles step by step.

| 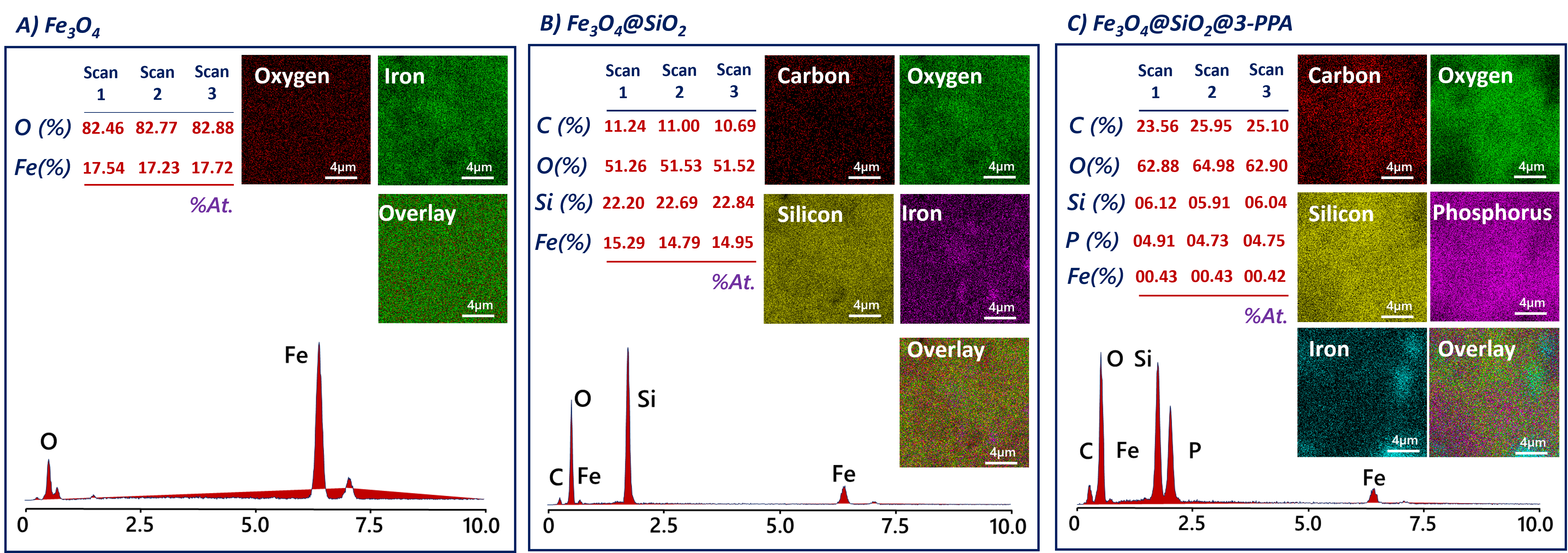 |
| --- |
| **Fig. SI-5.** EDS and EDS-Mapping of Fe_3_O_4_, Fe_3_O_4_@SiO_2_, and Fe_3_O_4_@SiO_2_@3-PPA particles. |

| 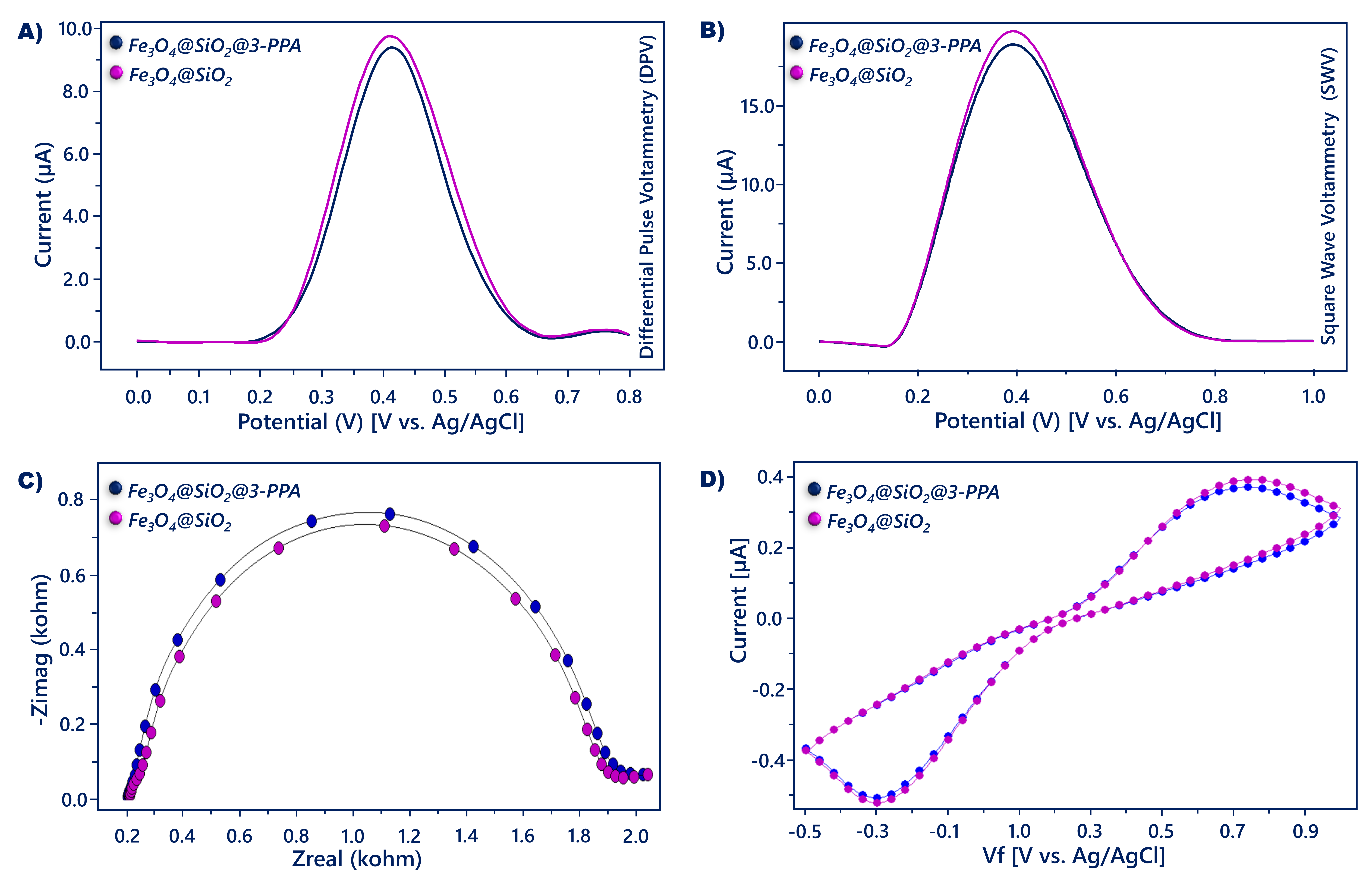 |
| --- |
| **Fig. SI-6.** DPV (A), SWV (B), EIS (C), and CV (D) analysis results for Fe_3_O_4_@SiO_2_ and Fe_3_O_4_@SiO_2_@3-PPA. |

**References**

1. Kong D, Yu M, Lin C, Liu X, Lin J, Fang J. (2005) Sol-gel synthesis and characterization of Zn_2_SiO_4_: Mn@ SiO_2_ spherical core-shell particles. J. Electrochem. Soc. 152(9):H146. https://doi.org/10.1149/1.1990612

2. Zhang T, Ge J, Hu Y, Zhang Q, Aloni S, Yin Y. (2008) Formation of hollow silica colloids through a spontaneous dissolution–regrowth process. Angew. Chem. 120(31):5890-5. https://doi.org/10.1002/ange.200800927

3. Jubb AM, Allen HC. (2010) Vibrational spectroscopic characterization of hematite, maghemite, and magnetite thin films produced by vapor deposition. ACS Appl. Mater. Interfaces. 2(10):2804-12. https://doi.org/10.1021/am1004943

4. Aydın M, Aydın EB, Sezgintürk MK. (2024) Label-free and ultrasensitive electrochemical cotinine sensing based on bio-modified magnetic nanoparticles. Sens. Actuators, B. 408:135476. https://doi.org/10.1016/j.snb.2024.135476
